# Alterations in the inferior longitudinal fasciculus in autism and associations with visual processing: a diffusion-weighted MRI study

**DOI:** 10.1186/s13229-018-0188-6

**Published:** 2018-02-08

**Authors:** Bart Boets, Lien Van Eylen, Kevin Sitek, Pieter Moors, Ilse Noens, Jean Steyaert, Stefan Sunaert, Johan Wagemans

**Affiliations:** 1 Center for Developmental Psychiatry, Department of Neurosciences, KU Leuven, Kapucijnenvoer 7h, PB 7001, 3000 Leuven, Belgium; 2 Leuven Autism Research (LAuRes), KU Leuven, 3000 Leuven, Belgium; 3 Department of Brain and Cognitive Sciences, Massachusetts Institute of Technology, Cambridge, MA 02139 USA; 4 Speech and Hearing Bioscience and Technology, Division of Medical Sciences, Harvard Medical School, Boston, MA 02115 USA; 5 Laboratory of Experimental Psychology, KU Leuven, 3000 Leuven, Belgium; 6 Parenting and Special Education Research Unit, KU Leuven, 3000 Leuven, Belgium; 7 Translational MRI, KU Leuven, 3000 Leuven, Belgium

**Keywords:** Autism spectrum disorder, Structural connectivity, Diffusion-weighted imaging, Visual processing, Inferior longitudinal fasciculus

## Abstract

**Background:**

One of the most reported neural features of autism spectrum disorder (ASD) is the alteration of multiple long-range white matter fiber tracts, as assessed by diffusion-weighted imaging and indexed by reduced fractional anisotropy (FA). Recent methodological advances, however, have shown that this same pattern of reduced FA may be an artifact resulting from excessive head motion and poorer data quality and that aberrant structural connectivity in children with ASD is confined to the right inferior longitudinal fasciculus (ILF). This study aimed at replicating the observation of reduced FA along the right ILF in ASD, while controlling for group differences in head motion and data quality. In addition, we explored associations between reduced FA in the right ILF and quantitative ASD characteristics, and the involvement of the right ILF in visual processing, which is known to be altered in ASD.

**Method:**

Global probabilistic tractography was performed on diffusion-weighted imaging data of 17 adolescent boys with ASD and 17 typically developing boys, matched for age, performance IQ, handedness, and data quality. Four tasks were administered to measure various aspects of visual information processing, together with questionnaires assessing ASD characteristics. Group differences were examined and the neural data were integrated with previously published findings using Bayesian statistics to quantify evidence for replication and to pool data and thus increase statistical power. (Partial) correlations were calculated to investigate associations between measures.

**Results:**

The ASD group showed consistently reduced FA only in the right ILF and slower performance on the visual search task. Bayesian statistics pooling data across studies confirmed that group differences in FA were confined to the right ILF only, with the evidence for altered FA in the left ILF being indecisive. Lower FA in the right ILF tended to covary with slower visual search and a more fragmented part-oriented processing style. Individual differences in FA of the right ILF were not reliably associated with the severity of ASD traits after controlling for clinical status.

**Conclusion:**

Our findings support the growing evidence for reduced FA along a specific fiber tract in ASD, the right ILF.

**Electronic supplementary material:**

The online version of this article (10.1186/s13229-018-0188-6) contains supplementary material, which is available to authorized users.

## Background

Autism spectrum disorder (ASD) is a neurodevelopmental disorder characterized by impairments in social reciprocity and communication, combined with restricted, repetitive and stereotyped patterns of behavior, interests or activities (RRBIs) [1]. Atypical sensory processing is also often reported and has been included in the new diagnostic RRBI criteria of ASD in DSM-5 [1]. Although the etiology of the disorder remains largely unknown, advanced genetic and neuroimaging studies point towards the involvement of altered brain connectivity [2], and the core behavioral and cognitive atypicalities have been related to reduced integration of information between different brain regions [3–5]. Reduced long-range connectivity has often been investigated in ASD, but there are many inconsistencies regarding the existence, the direction, and the specific anatomical location of this aberrant brain connectivity [6–10].

Here, we investigated structural brain connectivity in ASD using diffusion-weighted imaging (DWI). This non-invasive magnetic resonance imaging (MRI) technique indirectly assesses the structural properties and orientation of white matter tracts based on the diffusion of water molecules [11]. Typically, reduced diffusivity along the principal axis (i.e., axial diffusivity or AD) and increased diffusivity perpendicular to it (i.e., radial diffusivity or RD), resulting in a reduced directionality of diffusion (i.e., lower fractional anisotropy or FA), are considered indicative of reduced white matter integrity and thus reduced structural connectivity [11]. However, this interpretation might be misleading as the exact microstructural and macrostructural substrates of reduced FA are only partly understood (e.g., axonal density, axonal diameter, degree of myelination, homogeneity of axon orientation) [12, 13]. Therefore, in the present report, we will refer to the observed diffusion properties per se, without making inferences about white matter integrity.

Previous studies have observed widespread reductions of FA in individuals with ASD and have interpreted these findings as indicative of generally reduced white matter connectivity in ASD [8, 9]. A meta-analysis of diffusion imaging studies revealed four clusters with consistently lower FA in individuals with ASD [8]. The largest cluster was located in the right occipito-temporal region and extended from the inferior occipital and lingual gyrus into the fusiform and inferior temporal gyrus. Fiber tracking through this cluster pinpointed aberrant connectivity along the right inferior longitudinal fasciculus (ILF) in ASD [8].

Recent findings, however, revealed that differences in diffusion properties may be an artifact resulting from excessive head motion and poorer DWI data quality [14, 15]. Individuals with ASD may be more prone to greater head motion and the resulting DWI artifacts, as shown by Koldewyn and colleagues [16]. These authors applied diffusion-weighted imaging combined with global probabilistic tractography in school-aged children with ASD versus typically developing (TD) children and showed reduced FA in ASD along multiple white matter tracts. However, after carefully matching data quality and head motion parameters between both groups, all these effects disappeared, except for consistently reduced FA in one single tract, the right ILF [16]. This study thus challenged the hypothesis of widespread changes in FA-related structural connectivity in ASD and highlighted the importance of matching for data quality.

The ILF is a white matter association tract, extending from the occipital cortex into the anterior temporal lobe [17]. The right ILF connects several brain regions that are crucially involved in face processing (e.g., the occipital face area, the fusiform face area, the superior temporal sulcus, and the amygdala) [6], and lesions of the right ILF have been associated with face processing impairments [18]. Given the characteristic difficulties of individuals with ASD with processing faces [19–21], Koldewyn and colleagues [16] a priori hypothesized to observe structural abnormalities in the right ILF in individuals with ASD. Yet, as the ILF (both right and left) carries information from many extrastriate visual areas throughout the ventral visual stream, it is also implicated in visual perceptual organization and object recognition in general [17, 22]. Therefore, alterations in the ILF may (at least partially) underlie the known visual processing anomalies of individuals with ASD. Although these perceptual atypicalities are often subtle and dependent on particular task and sample characteristics [23], it has been shown that individuals with ASD have problems with global integrative processing and are more inclined to process and attend to parts and details [24–26], as postulated by the Weak Central Coherence (WCC) account [24].

The overall aim of this study was threefold. First of all, we aimed to replicate the findings of Koldewyn and colleagues [16], obtained in children, in an independent sample of adolescents with ASD and TD controls, using an identical methodological approach. In particular, we expected individuals with ASD to show selectively reduced FA of the right ILF. Therefore, the right ILF constituted the main anatomical target, along with 17 other major white matter tracts that were also included in the study of Koldewyn and colleagues [16]. Second, we explored whether reduced FA of the right ILF is associated with increased ASD symptom severity, both regarding the social and the non-social (i.e., RRBI) domains. Given its involvement in face processing, which is crucial for efficient social communication and interaction, we mainly expected an association of ILF properties with the social symptom domain. Yet, the ILF also plays a role in visual perception [17, 22], and given that the perceptual peculiarities of individuals with ASD are proposed to underlie (at least some of) their RRBIs [27], it is warranted to also explore the association between ILF properties and general RRBI symptomatology. Third, given the involvement of the ILF in ventral visual stream processing, we examined the association between ILF diffusion properties and performance on several visual processing measures. Although both left and right ILF play a role in visual processing, within the context of studying an ASD sample and in line with our previous hypothesis, we mainly focused on the association between individual differences in FA of the right ILF and visual processing measures. Four tasks that are often used in ASD research and that each target visual information integration in a different manner were administered: a Fragmented Object Outlines task, a Coherent Motion task, a visual search task, and the Rey-Osterrieth Complex Figure task (ROCF) (for a detailed conceptual and technical description of these tasks, see Van Eylen et al. [23]). Both the Fragmented Object Outlines task and the Coherent Motion task require visual information integration. However, the Fragmented Object Outlines task relies more on ventral visual stream functioning, as it requires form integration [28, 29], whereas the Coherent Motion task relies on dorsal visual stream functioning, since it requires the integration of motion signals [30]. Therefore, it is expected that only performance on the Fragmented Object Outlines task is associated with ILF properties. The visual search task is more controversial in terms of the implicated visual processes. While it has traditionally been conceptualized to measure local processing abilities, it also requires various types of grouping and feature integration [31, 32] and may therefore also be associated with ventral stream ILF diffusion properties. Finally, the ROCF task does not provide an indication of processing abilities but provides an indication of processing style, with a higher score indicating a more fragmented, locally oriented processing style, and a lower score reflecting a more global integrative processing style [23]. For this task, we expect that a more integrative processing style is related to higher FA in the ILF.

## Methods

### Participants

Nineteen boys with ASD and 19 TD boys participated in the study. All participants were aged between 11 and 18 years, had a full-scale IQ (FSIQ) above 80 and had normal or corrected-to-normal vision (with glasses or lenses). Data on pubertal development were not collected. Participants were excluded if they had a history of epilepsy, traumatic brain injury, and attention deficit/hyperactivity disorder or if ASD was associated with a genetic syndrome. One individual with ASD had dyslexia and one had a developmental coordination disorder. None of the participants took psychotropic medication. Inclusion criteria for the ASD group were (1) a diagnosis of ASD made by the multidisciplinary Expertise Center for Autism (University Hospitals KU Leuven) in a standardized way according to DSM-IV-TR criteria [33]; (2) confirmation of their diagnosis with the Developmental, Dimensional, and Diagnostic interview (3di) [34] and (3) *T*-scores above 65 on the Social Responsiveness Scale (SRS) [35, 36]. None of the TD participants, nor their first degree relatives, had a history of neurological or psychiatric conditions, nor a current medical, developmental or psychiatric diagnosis. Parents of the control children completed the SRS questionnaire [35, 36] to exclude the presence of substantial ASD characteristics.

Application of a strict DWI data quality criterion (cf. supra) resulted in the selection of 17 adolescents with ASD (two left-handed) and 17 TD adolescents (two left-handed), matched for age, performance IQ, sex, handedness, and MRI data quality (see Tables 1 and 2). Both groups differed (marginally) significantly with regard to verbal and total IQ. However, as we aimed to replicate the study of Koldewyn and colleagues [16] as closely as possible, we ensured to control for the same participant characteristics as these authors (i.e., age, performance IQ, sex, and data quality measures).

**Table 1:** Participant characteristics

	ASD (*n* = 17)		TD (*n* = 17)		
	*M*	*SD*	*M*	*SD*	*p value*
Age (years)	13.8	1.3	14.4	2.0	.27
Performance IQ^a^	104	15	112	15	.14
Verbal IQ^a^	105	18	116	13	.05
Total IQ^a^	105	14	114	10	.03
Social Responsiveness Scale (Total *T*)^b^	90	10	44	8	< .0001
SRS Social Communication and Interaction	78	15	14	10	< .0001
SRS RRBI	16	5	1.3	1.6	< .0001
Repetitive Behavior Scale—Revised	21	11	0.5	1.3	< .0001

^a^Standardized scores with population average *M* = 100 and *SD* = 15

^b^Standardized scores with population average *M* = 50 and *SD* = 10

**Table 2:** Between-group differences for DWI data quality measures and for FA per tract

	ASD (*n* = 17)		TD (*n* = 17)		*F*	*p* value
	*M*	*SD*	*M*	*SD*		
DWI data quality measures						
Average translation	1.4279	0.3591	1.4271	0.3597	0.00	0.995
Average rotation	0.0130	0.0047	0.0114	0.0041	1.16	0.290
Percentage of slices with drop-out	0.2781	0.4457	0.1903	0.6030	0.23	0.633
Average signal drop-out score	1.0381	0.0437	1.0187	0.0332	2.12	0.155
Fractional anisotropy (FA) per tract						
R inferior longitudinal fasciculus ^*,°^	0.4395	0.0259	0.4622	0.0293	5.74	0.023
L inferior longitudinal fasciculus ^*^	0.4561	0.0319	0.4779	0.0286	4.43	0.043
R anterior thalamic radiations	0.3900	0.0261	0.4012	0.0208	1.94	0.173
L anterior thalamic radiations	0.3928	0.0248	0.4033	0.0295	1.26	0.269
R cingulum-angular bundle	0.3131	0.0351	0.2983	0.0215	2.21	0.147
L cingulum-angular bundle	0.2953	0.0379	0.2880	0.0303	0.38	0.541
R cingulum-cingulate gyrus bundle	0.4161	0.0373	0.4302	0.0527	0.81	0.374
L cingulum-cingulate gyrys bundle	0.4492	0.0468	0.4725	0.0596	1.61	0.214
R corticospinal tract	0.5243	0.0196	0.5228	0.0397	0.02	0.888
L corticospinal tract	0.5352	0.0230	0.5197	0.0307	2.79	0.105
R superior longitudinal fasciculus—parietal	0.4037	0.0253	0.4103	0.0263	0.56	0.461
L superior longitudinal fasciculus—parietal	0.4234	0.0485	0.4300	0.0381	0.19	0.662
R superior longitudinal fasciculus—temporal	0.4711	0.0271	0.4816	0.0270	1.30	0.263
L superior longitudinal fasciculus—temporal	0.4642	0.0304	0.4714	0.0274	0.52	0.477
R uncinate fasciculus	0.4002	0.0247	0.4018	0.0276	0.03	0.862
L uncinate fasciculus	0.3810	0.0247	0.3855	0.0244	0.28	0.603
Forceps major (corpus callosum)	0.5252	0.0866	0.5326	0.0759	0.07	0.793
Forceps minor (corpus callosum)	0.4976	0.0329	0.4938	0.0329	0.11	0.737

Note. ^*^p < .05, ^°^group difference that survives Bonferroni correction (α = 0.05/18 = 0.0028), after outlier exclusion (*F*(1,30) = 12.40, *p* = .0014)

The study was approved by the local Ethical Board and informed consent was obtained from all parents/guardians according to the Declaration of Helsinki, with additional assent from all participating children.

### DWI data acquisition

After familiarization in a mock scanner, scanning was performed with a 32 head coil 3T Philips Achieva system at the University Hospitals Leuven. DWI data covering the entire brain and brainstem were acquired using an optimized single-shot spin-echo, echo planar imaging sequence with the following parameters: 58 contiguous saggital slices, slice thickness = 2.5 mm, repetition time (TR) = 7600 ms, echo time (TE) = 65 ms, field-of-view (FOV) = 240 × 200 × 145 mm^2^, matrix size = 96 × 94, in-plane pixel size = 2.12 × 2.5 mm^2^, acquisition time = 10 min 33 s. Diffusion gradients were applied in 60 non-collinear directions (*b* = 1300 s/mm^2^) and one image without diffusion-weighting was acquired. Additionally, a high-resolution *T*_*1*_-weighted anatomical scan was collected (182 contiguous coronal slices, TR = 9.6 ms, TE = 4.6 ms, FOV = 250 × 250 × 218 mm^3^, acquisition matrix = 256 × 256, voxel size = 0.98 × 0.98 × 1.2 mm^3^, acquisition time = 6 min 23 s).

### DWI data processing

**Fig. 1:**
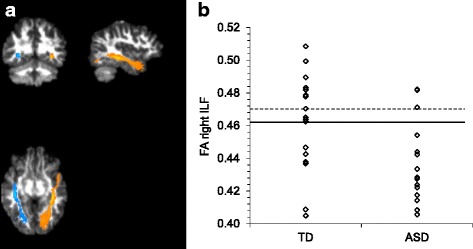
**a** Illustrative result of the left (in blue) and right (in orange) ILF pathway reconstructed by TRACULA for one representative subject, plotted on its DWI FA map. **b** Individual FA scores for the right ILF for TD and ASD participants. The solid line indicates the mean of the TD group. The dotted line indicates the mean of the TD group after exclusion of the two outliers

DWI data were analyzed using anatomically constrained global probabilistic tractography combined with an extensive data quality control, identical to the approach pursued by Koldewyn and colleagues [16]. Tractography was carried out using the Tracts Constrained by Underlying Anatomy (TRACULA) tool within FreeSurfer [37] (Fig. 1a). TRACULA is a tool for automatic reconstruction of 18 major white matter pathways in native subject-space from diffusion-weighted MR images. It uses global probabilistic tractography with anatomical priors. Prior distributions on the neighboring anatomical structures of each pathway are derived from an atlas and combined with the FreeSurfer cortical parcellation and subcortical segmentation of each subject’s T1 structural image [38] to constrain the tractography solutions. The posterior distribution of each of the white matter pathways is modeled as the product of (1) a data likelihood term, which uses the ball-and-stick model of diffusion and (2) a pathway prior term, which incorporates prior anatomical knowledge about the pathway trajectory from a set of training subjects. There is no assumption that the pathways have the same shape in the study subjects as in the training subjects, and thus, TRACULA does not rely on perfect alignment between study and training subjects.

Automated parcellation of the *T*_*1*_-weighted images was performed with FreeSurfer 5.3.0 to identify gray and white matter volumes and to define specific cortical and subcortical regions in each participant [39]. All preprocessing of the diffusion-weighted images (image corrections, image quality assessment, intra-subject and inter-subject-registration, mask creation, tensor fitting, estimation of pathway priors), ball-and-stick model fitting, pathway reconstruction, and extraction of DWI statistics were done using standard TRACULA settings. For each of the reconstructed tracts, mean values for FA, mean diffusivity (MD), RD, and AD were calculated by averaging the voxel values along the entire tract. In addition, supplementary analyses were performed where we calculated the DWI statistics only for the center of each tract (i.e., the single-voxel wide path with the highest probability, along the entire tract) or where we calculated a weighted tract average by weighting each DWI measure at each voxel in the tract by the pathway probability at that voxel.

DWI data quality measures comprised the average volume-by-volume translation, the average volume-by-volume rotation, the percentage of slices with excessive intensity drop-out, and the average drop-out score for slices with excessive intensity drop-out [40]. Subjects exceeding an average translation of 2.5 mm and/or rotation of 1.5° were discarded from the sample. This head motion criterion resulted in the removal of two ASD and two TD participants, and resulted in two participant groups that were well matched in terms of the four motion characteristics (see Table 2).

### Visual processing measures and behavioral questionnaires

Prior to scanning, all participants performed four visual processing tasks, as part of a study of Van Eylen and colleagues [23]. In the *Fragmented Object Outlines* task, the outline of an object was gradually built up in ten steps, from the most fragmented image (showing 10% of the contour) to the completely closed contour, and participants had to correctly identify the object as soon as possible. The main outcome measure was the correct identification latency (in ms), with a higher score reflecting slower performance. This task requires bottom-up contour integration, as well as top-down matching of the perceptual input with object representations stored in memory, and semantically labeling it. In the *Coherent Motion* task, participants were presented with a random dot kinematogram and had to detect the direction of coherently moving dots by integrating the motion stimuli. A coherent motion threshold was estimated by varying the percentage of coherently moving dots. This threshold reflects the smallest proportion of coherently moving dots that is necessary to reliably perceive the global direction of motion, with higher scores reflecting reduced performance. In the *visual search* task, participants watched a stimulus display containing a pre-specified target hidden among distractors, and participants had to touch the target as soon as possible on a touch screen. Two within-subject factors were manipulated: the number of distractors (14 vs. 24) and the target-distractor similarity (low vs. high). The target detection latency (in ms) was registered, which is the time needed to touch the correct target. Finally, the *Rey-Osterrieth Complex Figure* (ROCF) task provided an indication of the visual processing style. Participants had to copy the ROCF, and the degree of continuity or coherence in the drawing process was evaluated by calculating a fragmentation score. This score ranged from 0 to 9, with a higher score indicating a more fragmented, locally oriented processing style, and a lower score reflecting a more global integrative processing style. A more detailed description of each of these tasks and their analysis approach is provided by Van Eylen and colleagues [23].

In addition to the experimental tasks, two questionnaires were administered to assess ASD characteristics. The *Social Responsiveness Scale* (SRS) assesses a wide range of behaviors characteristic of ASD and covers subscales for “social communication and interaction” and for “restricted and repetitive patterns of behavior and interests” (RRBIs) [36]. Likewise, the *Repetitive Behavior Scale—Revised* (RBS-R) assesses the RRBIs observed in individuals with ASD [41].

### Statistical analysis

Distribution analyses were performed and measures were log_10_ or square root transformed to obtain normal distributions. For the DWI data, two outliers were identified in the FA data of the right ILF in the TD group (cf. Fig. 1b). All analyses were performed with these outliers included as well as excluded, and we report the more valid analysis, i.e., including the outliers for the group comparison and excluding the outliers for the correlation analyses (since the correlation analyses were disturbed by these outliers). Concerning the group comparisons, standard ANOVAs were performed for the DWI measures, the SRS and RBS-R questionnaires, the Coherent Motion test, and the ROCF fragmentation score. For the Fragmented Objects Outline task, a repeated measures mixed model analysis was carried out with group (ASD vs. TD) as between-subject variable and stimulus type (curved vs. straight) and stimulus homogeneity (low vs. high) as within-subject variables. For the visual search task, a repeated measures mixed model analysis was carried out with group (ASD vs. TD) as between-subject variable and target-distractor similarity (low vs. high) and number of distractors (14 vs. 24) as within-subject variables. For the repeated measures analyses, the Kenward-Roger method was used to calculate the degrees of freedom, and group contrasts for specific levels of a within-subject factor were corrected using the Tukey-Kramer procedure. Regarding the correlation analyses, whole-sample (partial) Pearson correlations were calculated to investigate the association between FA values of the white matter tracts, ASD characteristics, and visual processing measures. All analyses were conducted using the general statistical software package SAS Version 9.4 [42].

All reported *p* values are uncorrected for multiple comparisons, except for a Tukey-Kramer correction for the post-hoc group contrasts in the repeated measures analyses (i.e., when comparing both groups on the low and high similarity condition of the visual search task). A Bonferroni correction for multiple comparisons was applied by dividing the significance level (*α* = 0.05) by the number of comparisons per type of analysis. For each type of analysis, we also report the Bonferroni corrected significance level (α) in the “Results” section. Note, however, that within the context of this replication study, we had a clear a priori hypothesis in which tract to observe group differences in diffusion properties, thus reducing the need to correct for multiple comparisons. Moreover, in a study with a relatively small sample and a large number of measures, correction for multiple comparisons would not only reduce the chance of making a type I error (i.e., incorrectly rejecting a null hypothesis, which results in false positives) but also dramatically enhance the chance of making a type II error (i.e., incorrectly accepting the null hypothesis, which results in false negatives) [43]. As studies in smaller samples are less sensitive to trivial effects and type I errors, correction for multiple comparisons is less relevant and scientific importance is better reflected by effect sizes and their confidence intervals [44]. Accordingly, Cohen’s *d* group effect sizes (and confidence intervals) were calculated by dividing the estimated group difference by the pooled standard deviation. To calculate the pooled standard deviation, a simplified formula could be used (√[(σ1^2^ + σ2^2^)/2]), because our samples have equal size. An effect size ranging from 0.2 to 0.3 is considered small, values around 0.5 are medium, and values of 0.8 or above are considered large effects [45].

Finally, to quantify the success or failure of our attempt to replicate the findings of Koldewyn and colleagues [16] concerning group differences in FA values for the 18 tracts, we performed three Bayes factor tests [46] (for the applied R-script, see Additional file 1). All these Bayes factors express the weighted likelihood ratio between a null hypothesis and an alternative hypothesis. Firstly, the *equality-of-effect-size Bayes factor* quantifies the evidence for the null hypothesis that the effect sizes in our study equal the effect sizes in the study of Koldewyn and colleagues [16], versus the alternative hypothesis that the effect sizes in both studies are not equal. For this Bayes factor, values higher than 1 indicate support for the null hypothesis (suggesting successful replication), whereas values lower than 1 indicate support for the alternative hypothesis. Secondly, the *replication Bayes factor* quantifies the evidence that the data provide for the hypothesis that the effect that we found in our replication attempt is consistent with the effect found in the original study of Koldewyn and colleagues [16], versus the null hypothesis that the effect is zero. Values higher than 1 indicate support for the replication hypothesis, whereas values lower than 1 indicate support for the null hypothesis. However, values between 3 and 1/3 are considered anecdotal and indicate that the outcome is indecisive [47]. This is the case when the difference between the postulated effect in the null hypothesis and the replication hypothesis is small, so when the effect found in the original study is non-significant and the effect size is close to zero. Therefore, this test is mainly relevant to evaluate the replication success of the significantly lower FA value in the right ILF for the ASD group, as reported by Koldewyn and colleagues [16]. Thirdly, we calculated the *fixed-effect meta-analysis Bayes factor*, in which we pooled the data from our study and the study of Koldewyn and colleagues [16]. This factor quantifies the evidence that the pooled data provide for the hypothesis that the true effect is present (i.e., the alternative hypothesis) versus absent (i.e., the null hypothesis), with values higher than 1 providing support for the alternative hypothesis. By pooling the data from both studies, we overcome the power problem of our current study. More specifically, with a sample size of 17 included participants per group, the population effect size needs to be 0.99 or higher, to achieve a power of 80%. Furthermore, only effect sizes of 0.69 or higher achieve a power of 50% and can thus result in a significant group difference (*p* < 0.05). We therefore lack power to detect more subtle group differences with an effect size smaller than 0.69. However, by pooling the data from both studies and by calculating the fixed-effect meta-analysis Bayes factor, we can quantify the combined evidence for the presence or absence of an effect for each of the 18 fiber tracts. For all Bayes factors (BFs), values in between 3 and 1/3 indicate that the data are ambiguous, making the outcome indecisive.

## Results

Statistics for the DWI data quality measures average volume-by-volume translation, average volume-by-volume rotation, percentage of slices with signal drop-out, and average signal drop-out severity are displayed in Table 2 and indicate that groups were well matched in terms of DWI data quality.

**Fig. 2:**
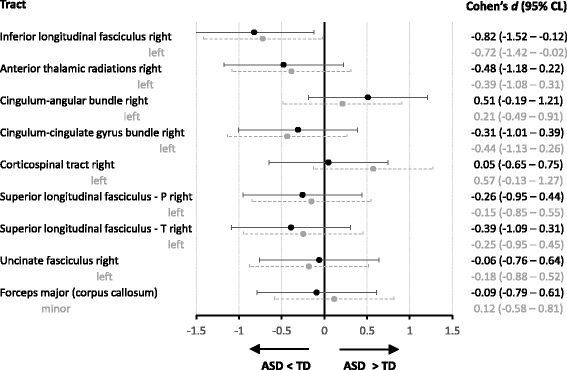
Effect sizes and 95% confidence limits (CL) for group differences in average FA per tract. Negative scores indicate a lower FA value in the ASD compared to the TD group. P, parietal; T, temporal

Given our aim to replicate the findings of Koldewyn and colleagues [16], we primarily focused on the FA values of the white matter tracts, especially of the right ILF. A one-way ANOVA comparing ASD vs. TD on each of the 18 tracts revealed similar FA values for both groups on every tract, with the exception of significantly reduced FA in ASD for the right and left ILF (see Table 2, and Figs. 1b and 2). To quantify the evidence that our data provide for replicating the findings from the study of Koldewyn and colleagues [16], we calculated three Bayes Factors (see Table 3). Firstly, the equality-of-effect-size Bayes factor was higher than 1 for all of the tracts, except for the right CAB, indicating that for all those tracts, there is more evidence that the effect sizes of both studies are equal. However, for several tracts, this Bayes factor was between 3 and 1/3 (i.e., for the left and right ATR, for the left CCG, CST and ILF, and for the right SLFT and CAB), indicating that the evidence for those tracts is indecisive (see Table 3). Secondly, the replication Bayes factor for the right ILF showed that it is 11.6 times more likely that the observed reduction in FA in the ASD group is a replication of the effect found in the study of Koldewyn and colleagues [16] than that this effect is truly zero. Interestingly, for the left ILF, the replication Bayes factor was also larger than 3 (i.e., 4.5). In line with our expectations, the replication Bayes factor was inconclusive (1/3 < BF < 3) for almost all other tracts (see Table 3), since the postulated effect under the replication hypothesis was close to that predicted under the null hypothesis. For the right CAB, however, this Bayes factor indicates that it is 3.8 times more likely that the difference in FA is truly zero than that we replicated the effect described by Koldewyn and colleagues [16]. Thirdly, the fixed-effect meta-analysis Bayes factor also provided strong evidence that the observed lower FA value in the right ILF for the pooled ASD group is a true effect (BF = 125). For the left ILF, the evidence is indecisive (BF = 2), but for all other tracts, this factor indicated that it is 6 to 23 times more likely that the effect is truly zero than that there is a group difference in FA (1/23.3 < BF < 1/6.3).

**Table 3:** An overview of the Bayes factors (BF) per tract to quantify the replication results [46]. For an explanation of each of these BFs, see the “Statistical analysis” section

	Equality-of-effect-size BF	Replication BF	Fixed-effect meta-analysis BF
R inferior longitudinal fasciculus	3.89	11.56	124.67
L inferior longitudinal fasciculus	2.69	4.45	2.00
R anterior thalamic radiations	1.93	0.95	0.05
L anterior thalamic radiations	2.77	1.00	0.05
R cingulum-angular bundle	0.54	0.26	0.12
L cingulum-angular bundle	4.08	1.00	0.16
R cingulum-cingulate gyrus bundle	3.33	0.97	0.12
L cingulum-cingulate gyrys bundle	2.58	1.11	0.14
R corticospinal tract	4.11	0.83	0.08
L corticospinal tract	1.56	1.15	0.04
R superior longitudinal fasciculus—parietal	3.32	0.89	0.06
L superior longitudinal fasciculus—parietal	3.42	0.73	0.09
R superior longitudinal fasciculus—temporal	2.85	1.07	0.13
L superior longitudinal fasciculus—temporal	3.97	1.02	0.15
R uncinate fasciculus	3.89	0.78	0.10
L uncinate fasciculus	3.81	0.86	0.06
Forceps major (corpus callosum)	4.18	0.87	0.10
Forceps minor (corpus callosum)	3.35	0.69	0.11

Concerning AD, RD, and MD, a one-way ANOVA comparing ASD vs. TD revealed similar values for both groups on each of the 18 tracts (all *p* > .10), with the exception of significantly increased RD in ASD in the right ILF (*F*(1,32) = 4.54, *p* = .041, *d* = .73).

Four supplementary analyses were performed on the DWI statistics. First, we calculated the DWI statistics only for the center of each tract, revealing reduced FA in ASD in right ILF (*F*(1,32) = 5.09, *p* = .031, *d* = − 0.77), marginally significantly reduced FA in ASD in left ILF (*F*(1,32) = 4.07, *p* = .052, *d* = − 0.69), and increased RD in ASD in right ILF (*F*(1,32) = 6.61, *p* = .015, *d* = 0.88), with all other measures similar for both groups (*p* > .10). Second, we calculated weighted DWI averages by weighting each DWI measure at each voxel in the tract by the pathway probability at that voxel. This analysis revealed reduced FA in ASD in right ILF (*F*(1,32) =.4.58, *p* = .040, *d* = − 0.73) and increased RD in ASD in right ILF (*F*(1,32) = 4.49, *p* = .042, *d* = 0.72), with all other measures similar for both groups (*p* > .098). Third, we reanalyzed the data of FA of right ILF after excluding two outlying TD subjects (as evidenced in Fig. 1b). This analysis revealed significantly reduced FA of the right ILF in ASD (*F*(1,30) = 12.40, *p* = .0014, *d* = − 1.25), which survives Bonferroni multiple comparison correction for the 18 assessed tracts (*α* = .05/18). Fourth, we reanalyzed the data of FA of the right ILF after excluding the four left-handed subjects, as handedness may reflect lateralization of language, which in turn may impact upon lateralization of face-sensitive areas [48]. This analysis again revealed significantly reduced FA of the right ILF in ASD (*F*(1,28) = 6.11, *p* = .019, *d* = − 0.90).

As expected, individuals with ASD scored significantly higher on each of the questionnaires assessing ASD characteristics, both for impairments in social communication and interaction and for RRBIs (cf. SRS, RBS-R; see Table 1). These results survive Bonferroni correction, since the *p* value is smaller than 0.0125 (*α* = 0.05/4).

Pertaining to the visual measures, individuals with ASD were slower to detect the target in the visual search task, particularly in the high-similarity condition with highly similar target-distractor items (see Table 4). When applying a stringent Bonferroni correction, this last group difference became marginally significant (*α* = 0.05/4 = 0.0125), although the effect size was medium to large (*d* = 0.72). No significant group differences were found on the other visual processing measures (i.e., the Fragmented Object Outlines task, the Coherent Motion task, and the ROCF task) (see Table 4).

**Table 4:** Between-group differences for the visual processing measures

	ASD (*n* = 17)		TD (*n* = 17)		Group test statistic	*p* value	Cohen’s *d*
	*M*	*SD*	*M*	*SD*			
Fragmented Objects Outline Task (ms) ^a^	4335	1252	4031	919	*F* = 0.91	.35	0.31
Coherent Motion Task (% coherence)	29	12	24	11	*F* = 2.02	.17	0.48
Visual search task (ms) ^b^	2075	505	1825	295	*F* = 2.93	.09	0.52
Low similarity ^c^	1792	421	1677	295	*t* = 0.72	.47	0.25
High similarity ^c^	2360	634	1973	381	*t* = 2.51	.017	0.72
ROCF fragmentation score	5.4	2.1	4.2	2.8	*F* = 1.76	.19	0.46

Note. Since the groups were compared on four tasks, a Bonferroni correction results in a significance level (*α*) of 0.0125

^a^Other effects retained in the model for Fragmented Objects Outline Task: within-subject factors type (*F*(1,1279) = 13.07, *p* < .001) and homogeneity (*F*(1,1279) = 54, *p* = < .001)

^b^Other effects retained in the model for visual search: within-subject factors target-distractor similarity (*F*(1,1285) = 86.07, *p* < .001), number of distractors (*F*(1,1285) = 57.67, *p* < .001), and the interaction between group and target-distractor similarity (*F*(1,1285) = 7.68, *p* = .006)

^c^Tukey-Kramer correction was performed for group contrasts for the specific levels of a within-subject factor

Next, we calculated whole-group Pearson correlations between FA of the right ILF and ASD characteristics and between FA of the right ILF and visual processing measures (see Additional files 2 and 3). As both groups were preselected to differ in terms of ASD symptoms, group membership was partialed out from all correlations involving ASD characteristics. While lower FA in the right ILF showed a slight association with the presence of more ASD characteristics (SRS total score: *partial r*(29) = − 0.27, *p* = .15; SRS Social Communication and Interaction scale: *partial r*(29) = − 0.26, *p* = .17; SRS RRBI scale: *partial r*(29) = − 0.18, *p* = .34), none of these correlations were significant, and the association only approached significance for the RBS-R questionnaire (*partial r*(29) = − 0.37, *p* = .05). Pertaining to the visual measures, lower FA in the right ILF showed a marginally significant association with slower visual search (*r*(29) = − 0.34, *p* = .059) and a more part-oriented processing style as indexed by the fragmentation score on the ROCF (*r*(30) = − 0.34, *p* = .05). However, these results did not survive Bonferroni correction (*α* = 0.006). No association was observed with coherent motion sensitivity (*r*(30) = − 0.23, *p* = .22) or performance on the Fragmented Object Outlines task (*r*(30) = − 0.23, *p* = .21). Due to the small sample size, none of these correlations sustained in the separate participant groups, except for associations in the ASD sample between lower FA in right ILF and a more fragmented processing style (ROCF: *r*(15) = − 0.50, *p* = .04) and a trend towards more RRBIs on the RBS-R questionnaire (*r*(14) = − 0.46, *p* = .07). In the TD group, the association with ASD characteristics was substantial but not significant (SRS: *r*(13) = −.42, *p* = .12). None of the 17 other white matter tracts showed an association with quantitative ASD characteristics or visual processing measures, except for two tracts: lower FA in the left and right cingulate-cingulum gyrus (CCG) bundle was associated with slower performance on the Fragmented Objects Outline task (*r*(30) = − 0.43, *p* = .01 and *r*(30) = − 0.35, *p* = .04, respectively) and with slower visual search (*r*(29) = − 0.45, *p* = .009 and *r*(29) = − 0.34, *p* = .049, respectively) (see Additional file 3). However, none of these associations survived Bonferroni correction (α = 0.006).

## Discussion

The literature on neural processing in ASD is extensive and characterized by divergent and inconsistent findings. This partially reflects the characteristic heterogeneity of the disorder [49], but may also be due to the use of suboptimal analysis approaches and less reliable data quality assessment. A recent study applied state-of-the-art global probabilistic tractography combined with stringent DWI data quality criteria and found that aberrant structural connectivity in children with ASD may be confined to one specific white matter tract, the right inferior longitudinal fasciculus or ILF [16]. To further consolidate this finding, we replicated the study design of Koldewyn and colleagues [16], by applying an identical analysis approach and identical head motion and DWI data quality matching, in adolescents with and without ASD.

Similar to the findings of Koldewyn and colleagues [16], we found that adolescents with ASD showed reduced FA in the ILF and not in any of the 16 other white matter tracts. In our study, significantly reduced FA in the right ILF was consistently observed across a number of different analysis approaches and the effect size was large (i.e., *d* = − 0.82 in the current adolescent sample, as compared to *d* = − 0.68, in the school-aged sample of Koldewyn and colleagues). Furthermore, the Bayes factor tests provided strong evidence that we replicated the findings of Koldewyn and colleagues [16] and that the observed lower FA value in the right ILF for the ASD group is a true effect. Likewise, in both studies, reduced FA in the right ILF was driven by increased RD in the ASD sample. The particular anatomical location of aberrant diffusion in ASD coincides with the major cluster of reduced FA across a series of whole-brain DWI studies (as calculated in a meta-analysis [8]) and is also supported by a number of DWI tractography studies [50–52]. Together with findings of reduced functional occipito-temporal connectivity [10], this suggests a dysfunction of the right ILF in ASD.

Contrary to previous studies and reviews [6, 8, 9, 16], we also observed significantly reduced FA in the left ILF (*d* = − 0.72) in the ASD group. However, this group difference was less consistently observed across different analysis approaches, and—as it was not a priori predicted—it did not survive correction for multiple comparisons. A closer look at the data of Koldewyn and colleagues [16] shows that FA in the left ILF was also significantly reduced in ASD in their original analysis without head motion and data-quality matching. Yet, in the more stringent analysis, the group difference was no longer significant, but still substantial (*d* = − 0.34). The replication Bayes factor also indicated that the observed lower FA value in the left ILF for the ASD group corresponds to the effect reported by Koldewyn and colleagues [16], rather than showing that the effect is zero. However, according to the fixed-effect meta-analysis Bayes factor, the pooled data of both studies provide indecisive evidence. Taken together, this indicates that the observation of reduced FA in the left ILF is not as robust as in the right ILF and should be interpreted with caution.

Concerning the issue of *selective* alterations in one particular white matter tract in ASD, it should be noted that our sample was too small to reliably demonstrate the *absence* of more subtle group differences in white matter properties. To overcome this limitation, we calculated the fixed-effect meta-analysis Bayes factor on the pooled data from our study and the study of Koldewyn and colleagues [16]. This Bayes factor provided strong evidence that the observed lower FA value in the right ILF of the ASD group is indeed a true effect (in fact, based on the findings in both studies, it is 125 times more likely that this effect is truly present instead of absent). For the left ILF, the evidence was inconclusive, but for all other tracts, the meta-analysis Bayes factor clearly suggested that group differences are absent (depending on the tract it is 6 to 23 times more likely that FA values across groups are similar instead of different). Therefore, the combined findings across both studies confirm the presence of alterations in right ILF in ASD and may question the common idea of more widespread alterations in white matter in ASD (although they leave us with uncertainty regarding the left ILF).

Additionally, our findings also illustrate the heterogeneity in white matter properties within the ASD population (see Fig. 1b), indicating that results of group comparisons should be interpreted with caution. This heterogeneity implies that not every individual participant with ASD has reduced FA in the right ILF, despite the observed group difference. Likewise, for the other tracts, the absence of a group difference in FA does not imply that none of the participants with ASD may show alterations in any of these tracts. Besides heterogeneity at the brain level [49], ASD is also characterized by heterogeneity at the cognitive level [53] and at the behavioral level, as each ASD symptom has a wide range of manifestations [1]. This heterogeneity at different levels stimulates a more dimensional approach to examine the link between alterations in white mater organization and variations in cognition and ASD symptom severity.

The observed alterations in white matter organization can potentially underlie some of the cognitive characteristics of ASD. In this study, we focused on the role of the right ILF in visual processing. According to one of the dominant theories on visual processing in ASD (i.e., the Weak Central Coherence account), individuals with ASD show relatively impaired global integrative processing and are more inclined to process and attend to parts or details [24]. Nevertheless, group differences in visual processing are often subtle, and many studies yield inconsistent results with weak effect sizes comparing individuals with ASD versus TD controls [25, 26]. The adolescents of our ASD sample showed reduced performance on the visual search task, compared to the TD group. Intact performance was found on the Fragmented Object Outlines task, the Coherent Motion task, and the ROCF. This pattern of results is in line with similar findings on the same visual measures in larger samples of individuals with and without ASD [23]. Van Eylen and colleagues [23] demonstrated that group differences on visual processing measures were small and depended on the age and/or sex of the participants. More specifically, the group difference on the ROCF task was only found in girls and the group difference on the Coherent Motion task was restricted to younger children, thus corresponding to the absence of group differences in the current sample of adolescent boys. Generally, these findings challenge the claim that individuals with ASD have a general inability to integrate information [23], although the cognitive heterogeneity within ASD should be taken into account when interpreting results.

Reduced brain connectivity has been suggested as the biological mechanism underlying atypical visual processing in individuals with ASD [4]. Here, we observed reduced FA in the right ILF of boys with ASD. As the ILF connects the occipital and the temporal lobe, reduced FA along this tract may impact upon information integration along the ventral visual stream. In line with this hypothesis, we found that lower FA in the right ILF was associated with slower performance on the visual search task and a trend towards a more part/detail-oriented processing style. Ortibus and colleagues [22] previously demonstrated that reduced FA of the ILF was also associated with impaired object recognition in children with cerebral visual impairment. As expected, FA in the ILF was not associated with Coherent Motion sensitivity, a traditional measure of dorsal visual stream functioning [30]. Contrary to our expectations, no correlation was found between FA in the ILF and performance on the Fragmented Object Outlines task, and no group difference was found on this task, despite the lower FA values in the right ILF of the ASD group. A possible explanation is that the Fragmented Object Outlines task targets higher-level top-down visual processing and, thus, does not solely rely on diffusion along the ILF. Therefore, specific alterations of the ILF may only partially determine performance on this task, due to compensation by other brain mechanisms. For example, our data pointed towards a possible involvement of the cingulum-cingulate gyrus bundle (CCG), given the observed association between FA in the CCG (bilaterally) and performance on the Fragmented Object Outlines task. Low- and mid-level visual processing tasks in which the right ILF plays a more unique role are expected to show a higher correlation with FA in the right ILF and a greater impairment in individuals with ASD.

One cognitive process in which the right ILF is particularly implicated is face processing, by interconnecting several key brain regions (i.e., the occipital and the fusiform face area, the superior temporal sulcus and the amygdala) [6, 18, 54–57]. Impaired (emotional) face processing has repeatedly been described in ASD [19–21] and may impact upon social functioning and communication in general. As a result, disruption of the right ILF may be linked with ASD symptom severity via face processing impairments. However, after controlling for clinical status, we could not observe any reliable associations between individual differences in FA of right ILF and individual differences in socio-communicative ASD characteristics. On the other hand, there was a marginally significant association between lower FA of the right ILF and the increased presence of RRBI characteristics as rated on the RBS-R questionnaire, which was entirely driven by the ASD sample. As some of these RBS-R items involve atypical sensory processing and a possible preoccupation with parts of objects, the link with altered ILF properties may be partially mediated by atypical visual processing [27].

Although this study replicated the previously observed reduction in FA in the right ILF in individuals with ASD [16], some limitations should be considered. First, as indicated above, by itself, our study was underpowered to demonstrate the selectivity of the ILF alterations in ASD, i.e., that *only* the right ILF and no other tracts may display reduced FA in ASD. Likewise, the power of our study was limited to demonstrate reliable associations with visual processing and ASD characteristics, especially at the subgroup level. Therefore, larger studies are needed to examine these associations more thoroughly, including the association with face processing abilities. Second, although both our study and the one of Koldewyn and colleagues [16] demonstrated reduced FA in right ILF in adolescents and school-aged children with ASD, respectively, it remains to be validated whether this ILF alteration is consistently present across the developmental trajectory, thus also at preschool and adult age. It may be particularly relevant to investigate how group differences in diffusion properties of association tracts evolve throughout pubertal development and how this relates to differences in hormone levels, such as testosterone. In this regards, it has been demonstrated that the development of association tracts, such as the ILF, is influenced by this hormone [58, 59] and that boys with and without ASD have different testosterone levels during puberty [60]. Third, the present study (as well as the one of Koldewyn and colleagues [16]) included a relatively selective subset of participants in terms of IQ (above 80), calling into question to what extent the findings may generalize to the whole ASD spectrum.

In this regard, future research should also investigate how heterogeneity at the behavioral level in the ASD population (in terms of IQ, age, comorbid symptoms etc.) may relate to heterogeneity at the neural level [61]. Future research is also needed to directly examine the hypothesized association between structural ILF properties and face processing abilities and to investigate (the directionality of) possible causal pathways linking lower FA in right ILF with ASD symptom severity. A longitudinal investigation of the intrinsic association between fine-grained local connectivity patterns and (atypical) functional brain activity in the fusiform face area [62] may be particularly elucidating in this regard. Finally, more fundamental research is needed to pinpoint the exact micro- and macrostructural factors underlying the reduced FA in the right ILF in individuals with ASD and the functional implications in terms of brain connectivity, neural communication, and information transmission.

## Conclusion

To conclude, our results replicate the findings of Koldewyn and colleagues [16] and support the growing evidence for altered structural connectivity along the right ILF in ASD, although they leave us with uncertainty regarding alterations in the left ILF. Nevertheless, these findings need to be interpreted in the light of the known heterogeneity of the disorder. This heterogeneity calls for a more dimensional approach to examine the link between alterations in ILF properties and variations in cognition and ASD symptom severity. In that regard, this study suggests that alterations in structural ILF properties in individuals with ASD may underlie (at least some of) the visual processing atypicalities and RRBI characteristics of ASD. To move the field forward, we need large interdisciplinary, multi-dimensional studies that examine inter-individual differences at different levels and the corresponding biological pathways. This will increase our understanding of the links between the brain, cognition, and behavior and will reveal the factors that induce, or at least increase the risk for, ASD, but may also elucidate protective factors.

## Additional files

Additional file 1: Supplementary R-script. The applied R-script to calculated the three Bayes factor tests, to quantify the result of our replication attempt [46]. (TXT 4 kb)

Additional file 2: Figure S1. Scatter plots displaying the association between individual differences in FA in right ILF (depicted on the *X* axis) and individual differences in quantitative ASD characteristics and visual processing measures (depicted on the *Y* axis). First row: Associations with (square root transformed) scores on the SRS questionnaire, the SRS Social and Communication subscale, the SRS RRBI subscale, and the RBS-R questionnaire. Second row: Associations with reaction time on the Fragmented Object Outlines task, (log-transformed) percentage coherence threshold on the Coherent Motion Task, (log-transformed) reaction time on the visual search task, and (square root transformed) fragmentation score on the Rey-Osterrieth Complex Figure task. ASD subjects are depicted by empty squares, TD subjects by filled diamonds. (PPTX 130 kb)

Additional file 3: Table S1. Pearson (partial) correlations between ASD characteristics, visual processing measures and fractional anisotropy (FA) in the white matter tracts. (DOCX 19 kb)

## Abbreviations

AD: Axial diffusivity
ASD: Autism spectrum disorder
DFlex: Detail and flexibility questionnaire
DWI: Diffusion-weighted Imaging
FA: Fractional anisotropy
FOV: Field-of-view
ILF: Inferior longitudinal fasciculus
MD: Mean diffusivity
MRI: Magnetic resonance imaging
RBS-R: Repetitive Behavior Scale—Revised
RD: Radial diffusivity
ROCF: Rey-Osterrieth Complex Figure
RRBIs: Restricted and repetitive patterns of behavior and interests
SRS: Social Responsiveness Scale
TD: Typically developing
TE: Echo time
TR: Repetition time
TRACULA: Tracts Constrained by Underlying Anatomy
