# Supplementary material for: Alterations in the inferior longitudinal fasciculus in autism and associations with visual processing: a diffusion-weighted MRI study
Source: Mol Autism. 2018 Feb 8;9:10. doi: 10.1186/s13229-018-0188-6 (PMC5806238; doi:10.1186/s13229-018-0188-6)
Supplement: Supplementary file 2 — Scatter plots displaying the association between individual differences in FA in right ILF (depicted on the X axis) and individual differences in quantitative ASD characteristics and visual processing measures (depicted on the Y axis). First row: Associations with (square root transformed) scores on the SRS questionnaire, the SRS Social and Communication subscale, the SRS RRBI subscale, and the RBS-R questionnaire. Second row: Associations with reaction time on the Fragmented Object Outlines task, (log-transformed) percentage coherence threshold on the Coherent Motion Task, (log-transformed) reaction time on the visual search task, and (square root transformed) fragmentation score on the Rey-Osterrieth Complex Figure task. ASD subjects are depicted by empty squares, TD subjects by filled diamonds. (PPTX 130 kb) [file 13229_2018_188_MOESM2_ESM.pptx]

## Slide 1
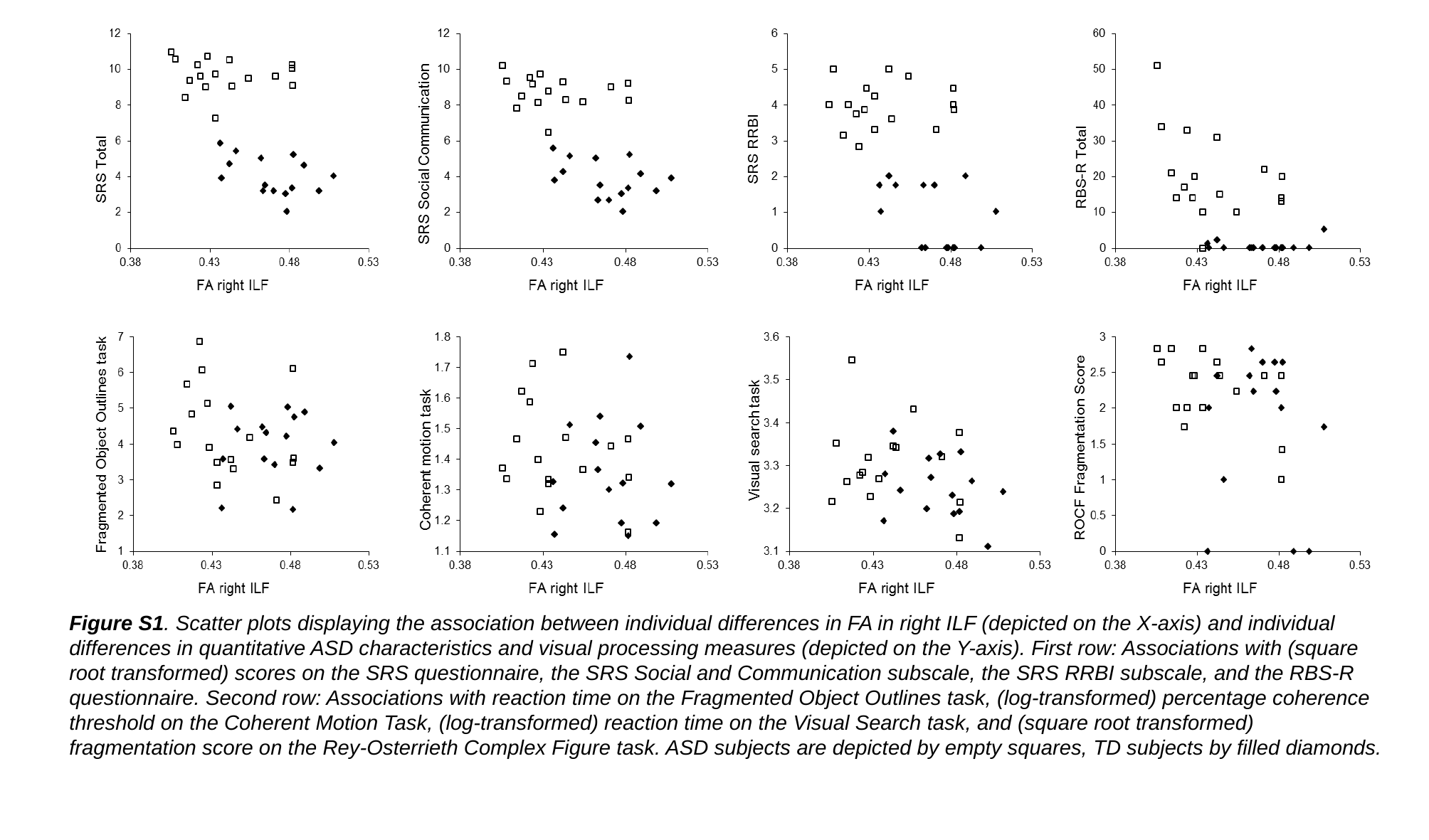

Figure S1. Scatter plots displaying the association between individual differences in FA in right ILF (depicted on the X-axis) and individual differences in quantitative ASD characteristics and visual processing measures (depicted on the Y-axis). First row: Associations with (square root transformed) scores on the SRS questionnaire, the SRS Social and Communication subscale, the SRS RRBI subscale, and the RBS-R questionnaire. Second row: Associations with reaction time on the Fragmented Object Outlines task, (log-transformed) percentage coherence threshold on the Coherent Motion Task, (log-transformed) reaction time on the Visual Search task, and (square root transformed) fragmentation score on the Rey-Osterrieth Complex Figure task. ASD subjects are depicted by empty squares, TD subjects by filled diamonds.
