# Supplementary material for: Alterations in the inferior longitudinal fasciculus in autism and associations with visual processing: a diffusion-weighted MRI study
Source: Mol Autism. 2018 Feb 8;9:10. doi: 10.1186/s13229-018-0188-6 (PMC5806238; doi:10.1186/s13229-018-0188-6)
Supplement: Supplementary file 3 — Pearson (partial) correlations between ASD characteristics, visual processing measures and fractional anisotropy (FA) in the white matter tracts. (DOCX 19 kb) [file 13229_2018_188_MOESM3_ESM.docx]

Additional file 3

Table S1

*Pearson correlations between ASD characteristics, visual processing measures and fractional anisotropy (FA) in the white matter tracts*

|  | ASD characteristics | | | | Visual processing measures | | | |
| --- | --- | --- | --- | --- | --- | --- | --- | --- |
| Fractional anisotropy per tract | SRS Total | SRS SCI | SRS RRBI | RBS-R Total | Fragmented Object Outlines | Coherent Motion | Visual Search | ROCF |
| R inferior longitudinal fasciculus | -0,57*** | -0,57*** | -0,552*** | -0,60*** | -0,23 | -0,23 | -0,34° | -0,34° |
| L inferior longitudinal fasciculus | -0,29 | -0,29 | -0,29 | -0,33° | -0,17 | -0,24 | -0,18 | -0,13 |
| R anterior thalamic radiations | -0,22 | -0,21 | -0,23 | -0,32° | -0,28 | -0,12 | -0,19 | -0,25 |
| L anterior thalamic radiations | -0,18 | -0,17 | -0,22 | -0,26 | -0,25 | -0,05 | -0,17 | 0,05 |
| R cingulum-angular bundle | 0,22 | 0,21 | 0,24 | 0,11 | -0,13 | 0,16 | 0,09 | -0,01 |
| L cingulum-angular bundle | 0,08 | 0,06 | 0,16 | -0,15 | -0,06 | -0,21 | 0,32 | -0,01 |
| R cingulum-cingulate gyrus bundle | -0,12 | -0,11 | -0,18 | -0,06 | -0,35* | -0,16 | -0,34* | -0,22 |
| L cingulum-cingulate gyrus bundle | -0,20 | -0,19 | -0,28 | -0,14 | -0,44* | -0,24 | -0,45** | -0,33° |
| R corticospinal tract | 0,01 | 0,02 | -0,05 | -0,02 | -0,10 | 0,03 | -0,20 | -0,12 |
| L corticospinal tract | 0,13 | 0,11 | 0,17 | 0,13 | -0,25 | 0,11 | -0,04 | 0,18 |
| R superior longitudinal fasciculus – parietal | -0,17 | -0,16 | -0,21 | -0,31 | -0,13 | -0,24 | -0,24 | -0,04 |
| L superior longitudinal fasciculus – parietal | -0,07 | -0,07 | -0,13 | -0,16 | -0,18 | -0,19 | -0,30 | -0,10 |
| R superior longitudinal fasciculus – temporal | -0,20 | -0,20 | -0,26 | -0,14 | -0,15 | -0,17 | -0,28 | -0,09 |
| L superior longitudinal fasciculus – temporal | -0,13 | -0,12 | -0,20 | -0,09 | -0,18 | -0,08 | -0,25 | -0,06 |
| R uncinate fasciculus | -0,07 | -0,07 | -0,09 | -0,15 | -0,22 | -0,11 | -0,22 | -0,19 |
| L uncinate fasciculus | -0,12 | -0,12 | -0,17 | -0,06 | -0,21 | 0,04 | -0,06 | 0,03 |
| Forceps major | -0,01 | -0,01 | -0,06 | 0,001 | -0,32° | -0,08 | 0,01 | -0,10 |
| Forceps minor | 0,05 | 0,03 | 0,06 | 0,04 | 0,06 | 0,18 | 0,17 | 0,23 |

° p < 0.07, * p < 0.05, ** p < 0.01, *** p < 0.006 surviving Bonferroni correction for multiple comparisons (α = 0.05/8)

SRS: Social Responsiveness Scale, SCI: Social Communication and Interaction scale, RRBI: Restricted and Repetitive patterns of Behavior and Interest scale, RBS-R: Repetitive Behavior Scale-Revised, ROCF: Rey-Osterrieth Complex Figure, R: right, L: left
